# Impaired hippocampal neurogenesis in vitro is modulated by dietary-related endogenous factors and associated with depression in a longitudinal ageing cohort study

**DOI:** 10.1038/s41380-022-01644-1

**Published:** 2022-07-07

**Authors:** Andrea Du Preez, Sophie Lefèvre-Arbogast, Raúl González-Domínguez, Vikki Houghton, Chiara de Lucia, Dorrain Y. Low, Catherine Helmer, Catherine Féart, Cécile Delcourt, Cécile Proust-Lima, Mercè Pallàs, Alex Sánchez-Pla, Mireia Urpi-Sardà, Silvie R. Ruigrok, Barbara Altendorfer, Ludwig Aigner, Paul J. Lucassen, Aniko Korosi, Claudine Manach, Cristina Andres-Lacueva, Cécilia Samieri, Sandrine Thuret

**Affiliations:** 1 Department of Basic and Clinical Neuroscience, Maurice Wohl Clinical Neuroscience Institute, Institute of Psychiatry, Psychology and Neuroscience, King’s College London, London, SE5 9NU UK; 2 University of Bordeaux, Inserm, Bordeaux Population Health Research Center, UMR 1219, F-33000 Bordeaux, France; 3 Nutrition, Food Science and Gastronomy Department, Faculty of Pharmacy and Food Science, University of Barcelona, 08028 Barcelona, Spain; 4 CIBER Fragilidad y Envejecimiento Saludable (CIBERFES), Instituto de Salud Carlos III, 0828 Barcelona, Spain; 5 Université Clermont Auvergne, INRA, UMR1019, Human Nutrition Unit, F-63000 Clermont Ferrand, France; 6 Pharmacology Section, Department of Pharmacology, Toxicology and Medicinal Chemistry, Faculty of Pharmacy and Food Sciences, and Institute of Neurosciences, University of Barcelona, Av. Joan XXIII, 27-31, E-08028 Barcelona, Spain; 7 Brain Plasticity Group, Swammerdam Institute for Life Sciences, Center for Neuroscience, University of Amsterdam, 1098 XH Amsterdam, The Netherlands; 8 Institute of Molecular Regenerative Medicine, Spinal Cord Injury and Tissue Regeneration Center Salzburg, Paracelsus Medical University, Salzburg, 5020 Austria; 9 Department of Neurology, University Hospital Carl Gustav Carus, Technische Universität Dresden, 01307 Dresden, Germany

**Keywords:** Neuroscience, Stem cells, Depression

## Abstract

Environmental factors like diet have been linked to depression and/or relapse risk in later life. This could be partially driven by the food metabolome, which communicates with the brain via the circulatory system and interacts with hippocampal neurogenesis (HN), a form of brain plasticity implicated in depression aetiology. Despite the associations between HN, diet and depression, human data further substantiating this hypothesis are largely missing. Here, we used an in vitro model of HN to test the effects of serum samples from a longitudinal ageing cohort of 373 participants, with or without depressive symptomology. 1% participant serum was applied to human fetal hippocampal progenitor cells, and changes in HN markers were related to the occurrence of depressive symptoms across a 12-year period. Key nutritional, metabolomic and lipidomic biomarkers (extracted from participant plasma and serum) were subsequently tested for their ability to modulate HN. In our assay, we found that reduced cell death and increased neuronal differentiation were associated with later life depressive symptomatology. Additionally, we found impairments in neuronal cell morphology in cells treated with serum from participants experiencing recurrent depressive symptoms across the 12-year period. Interestingly, we found that increased neuronal differentiation was modulated by increased serum levels of metabolite butyrylcarnitine and decreased glycerophospholipid, PC35:1(16:0/19:1), levels – both of which are closely linked to diet – all in the context of depressive symptomology. These findings potentially suggest that diet and altered HN could subsequently shape the trajectory of late-life depressive symptomology.

## Introduction

Major depressive disorder (MDD) is a debilitating condition that significantly impacts upon the physical, emotional and social wellbeing of individuals and their relatives [1]. Moreover, MDD is highly prevalent across all age ranges and consequently represents a major financial burden globally [2]. Given the devastating consequences of MDD, late-life depression, in particular, is an important public health concern, increasing the risk of morbidity and suicide, decreasing physical, cognitive and social functioning, and increasing self-neglect in later life - all of which are subsequently associated with increased mortality [3].

Importantly, late-life depression has also been consistently associated with an increased risk of cognitive decline (CD) and dementia [4, 5] both of which also significantly increase in risk in later life [6]. Furthermore, these conditions are often comorbid [7], and, therefore, treating one condition could consequently alleviate the associated symptoms of the other(s). Thus, late-life depression could also represent a target for preventing or alleviating CD and/or dementia [8].

Unfortunately, the development and implementation of effective pharmacological treatments for MDD is struggling to keep pace with the growth of its prevalence [9] and to better target MDD, we need to go beyond pharmacological intervention and seek other methods of modifying depression risk. One relevant avenue is environmental and lifestyle modification, in particular diet, which has been associated with depression in later life [10]. Indeed, several epidemiological studies have demonstrated how a higher adherence to the Mediterranean diet was associated with a reduced MDD prevalence in ageing populations [11, 12]. However, as yet it is unclear how exactly diet could influence depression outcomes on a biological level [13].

One relevant biological process associated with depression [14, 15] that is also modulated by diet [16, 17], is adult hippocampal neurogenesis (HN; the birth of new neurons derived from stem cells present in the hippocampus [18, 19]). Evidence to support an important role for HN in depression aetiology stems from research showing how chronic stress exposure (a risk factor for depression) supresses HN [20, 21], which, importantly, correlates with hippocampal volume and is notably required for antidepressant drugs to be effective in rodent models of depression [22, 23].

However, currently it is impossible to test the effects of diet on neurogenesis in live humans, and one option is to use an in vitro readout of human HN [24–27]. The main concept and relevance of this in vitro assay stems from the fact that the hippocampal neurogenic niche is in close vicinity to blood vessels, allowing direct communication with the systemic environment [28]. As such, HN is responsive to systemic and peripheral modulators like stress, inflammation and diet [16, 29] - all cues extrinsic to the brain and delivered via the blood. This idea is further supported from several lines of research. For example, Villeda and colleagues (2011), in their in vivo parabiosis model, demonstrate that ageing blood in young animals can indeed decrease HN [30, 31], while specific blood factors have been shown to transfer the beneficial effects of exercise on HN and cognition in rodents [32, 33]—all emphasising a role for modulation of HN by blood-borne factors.

Therefore, here we set out to study the effect of the systemic environment on the hippocampal neurogenic process in participants with and without depressive symptomology, using an in vitro cellular HN assay. Specifically, we used serum samples taken at inclusion of a longitudinal ageing cohort to: (i) determine whether changes in the neurogenic process are associated with depressive symptomology and chronicity across a 12-year period, (ii) explore the relationship between CD and depressive symptomology and the impact this may have on neurogenesis, and (iii) ascertain whether nutritional, metabolomic, and lipidomic biomarkers could modulate these HN outcomes.

## Methods and materials

### Cohort and study design

**Fig. 1:**
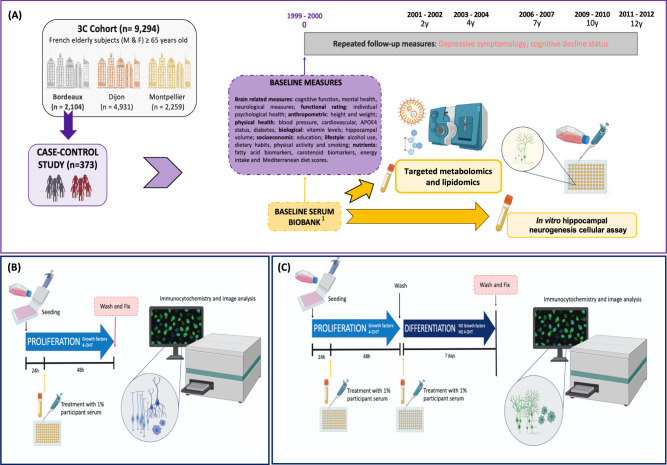
Cohort, study design and cellular assays. **A** **Three City (3C) cohort and study design:** The 3C cohort is a French population-based cohort that started in 1999–2000 and consists of male and female community dwellers aged >65 years (*n* = 9294). Participants from the 3C study were recruited from three French cities: Bordeaux (*n* = 2104), Dijon (*n* = 4931), and Montpellier (*n* = 2259), and specifically, a subsample nested within the 3C-Bordeaux cohort (*n* = 373) was used for this study. At baseline (0y), face-to-face interviews were conducted to collect sociodemographic and lifestyle characteristics, medical information, cognitive testing, blood pressure, and anthropometric measurements from all participants. Additionally, fasting blood samples were collected for constitution of a biobank; the serum samples of which were used for the metabolomics, lipidomics and the in vitro cellular assays, whereas the plasma samples were used to extract the nutritional biomarker data. Follow-up visits were performed every 2 to 3 years over a 12-year period and assessed depressive symptomology and cognitive decline. Cases were classified as all participants that reported high depressive symptomology (i.e., ≥17 in men and of ≥23 in women on the CES-D scale) at any timepoint (including at baseline) across the 12-year study period, whereas controls were all participants that did not report experiencing high depressive symptomology. **In vitro neurogenesis cellular assays:** **B** Proliferation assay: 24 h after seeding, cell medium was replaced with fresh medium containing 1% serum and 1:100 penicillin streptomycin (PenStrep; 10,000 U/mL) and was subsequently left to incubate for 72 h before being fixed in 4% paraformaldehyde (PFA), stained and proliferation specific markers quantified. **C** Differentiation assay: after 48 h of proliferation in the presence of 1% serum and 1:100 PenStrep (same as proliferation assay), cells were washed and treated with another serum supplementation, this time in medium absent of 4-hydroxytamoxifen (4-OHT) and growth factors: epidermal growth factor (EGF) and basic fibroblast growth factor (FGF), to allow cells to spontaneously differentiate. Serum-treated cells and were subsequently left to differentiate for a further 7 days before being fixed in 4% PFA, stained, and differentiation specific markers quantified. Cognitive decline status definition: Participants were classified as either cognitively stable or with accelerated cognitive decline based on their average performance in five neuropsychological tests (i.e., the Mini-Mental State Examination, the Benton Visual Retention Test, the Isaac’s Set Test, and the Trail-Making Test part A and part B) across five follow-up visits across the 12-year study duration. (1) Serum samples used for the metabolomics, lipidomics and in vitro assays are aliquots taken from the same batch. 3C three city, M male, F female, y years, h hours, 4-OHT 4-hydroxytamoxifen, CES-D Epidemiologic Studies Depression scale. Image created using BioRender software.

Serum samples were from participants of the Three-City (3C) cohort [34], specifically, from a case-control study on CD (*n* = 373) nested within the 3C-Bordeaux centre as described before [35]. Briefly, at baseline, fasting blood samples and sociodemographic, lifestyle and clinical measures were collected from all participants. Follow-up visits were performed every two to three years over 12 years during which depressive symptomology were assessed (Fig. 1A). The 3C study protocol was approved by the Consultative Committee for the Protection of Persons participating in Biomedical Research at Kremlin-Bicetre University Hospital (Paris, France). For further detail, see Table 1 and Supplementary Materials.

**Table 1:** Participant characteristics and in vitro neurogenesis readouts as stratified by depressive symptomology and chronicity (*n* = 373).

Values represent mean (SD) or *N* (%) of non-missing values. Characteristics (and associated values) in bold are covariates, all of which are controlled for in relevant models. # Also adjusted for in further analyses where relevant. FDR correction was applied to control for multiple testing. Cellular readouts expressed as a percentage relative to neural (DAPI) cell number. Cell line: HPC0A07/03; Passage number: P15-21; Technical replicates: *n* =3.

*ApoE-ε4* allele ε4 for the apolipoprotein E gene, *HDL* high-density lipoprotein, *LDL* low-density lipoprotein, *BMI* body mass index, *CVD* cardiovascular disease, *DAPI* 4′,6-diamidino-2-phenylindole, *SOX2* sex determining region Y (SRY)-box 2, *CC3* cleaved caspase 3, *DCX* doublecortin, *MAP2* microtubule-associated protein 2, *SD* standard deviation.

*p <  0.05; **p  <  0.01, ***p <  0.001.

^a^Estimated using logistic regressions controlling for age, gender, education, and cognitive decline status. Cognitive decline status definition: Participants were classified as either cognitively stable or with accelerated cognitive decline based on their average performance in five neuropsychological tests (i.e., the Mini-Mental State Examination, the Benton Visual Retention Test, the Isaac’s Set Test, and the Trail-Making Test part A and part B) across five follow-up visits across the 12-year study duration [35].

^b^Education was based on the highest level of attainment and considered dichotomously: either as no or primary level education only or as secondary/high school level and above.

^c^ApoE genotype was considered dichotomously: presence of at least one ε4 allele.

^d^Blood pressure ≥140/90 mmHg or antihypertensive medication use.

^e^Glucose ≥7.2 mmol/L or antidiabetic medication use.

^f^Fasting plasma total cholesterol ≥6.2 mmol/L or lipid-lowering medication use.

^g^History of cardiovascular or cerebrovascular disease.

^h^Includes all antihypertensive drugs, calcium channel blockers, diuretics, beta-blockers, and drugs acting on the renin-angiotensin system.

^i^Includes all antidiabetic drugs except insulin.

^j^Includes all statins, fibrates, or bile acid sequestrants.

^k^Includes all psycholeptics and psychoanaleptics—antidepressants, psychostimulants, and nootropics.

^l^Practice and intensity of physical exercise was assessed using a physical activity questionnaire for the elderly [100]. Regular exercise was classified as doing sport regularly or having at least one hour of leisure or household activity per day. Described in detail in [101].

### Depressive symptomology outcomes

Depressive symptomatology was evaluated using the Centre for Epidemiologic Studies Depression (CES-D) scale [36, 37]. Scores of ≥17 in men and of ≥23 in women were used as indicators of clinically relevant depressive symptomatology [38]. Additionally, to assess symptom chronicity, cases were further categorised based on whether a high depressive symptomology was detected once (i.e., single occurrence) or multiple times (i.e., recurrent symptoms) across the study. To retain a maximum number of participants with depressive symptomology, participants that had symptoms at baseline were retained in all analyses, and baseline depressive symptomology was controlled for in all analyses. For further detail, see Supplementary Materials.

### Nutritional variables

The concentrations of 23 nutritional biomarkers (i.e., 12 fatty acids, 6 carotenoids, 25(OH)D, alpha and gamma tocopherol, retinol, transthyretin) were determined in total plasma as previously described [39–41]. The metabolite and lipid data were extracted from serum using a large-scale, quantitative multi-metabolite platform and shotgun MS lipidomics, respectively, as described previously [42, 43]. For a full list of all nutritional variables, see Table 2.

**Table 2:** Associations between participant characteristics, nutritional data and altered in vitro neurogenesis readouts.

Participant characteristics and nutritional related data in bold are covariates, all of which are controlled for in relevant models. # Also adjusted for in further analyses where relevant. FDR correction was applied to control for multiple testing. Cell line: HPC0A07/03; Passage number: P15-21; Technical replicates: *n* = 3.

*ApoE-ε4* allele ε4 for the apolipoprotein E gene, *HDL* high-density lipoprotein, *LDL* low-density lipoprotein, *BMI* body mass index, *CVD* cardiovascular disease, *DAPI* 4′,6-diamidino-2-phenylindole, *SOX2* sex determining region Y (SRY)-box 2, *CC3* cleaved caspase 3, *DCX* doublecortin, *MAP2* microtubule-associated protein 2, *SD* standard deviation.

*p < 0 .05; **p  <  0.01; ***p  <  0.001.

^a^Estimated using linear regressions controlling for age, gender, education, and cognitive decline status. Cognitive decline status definition: Participants were classified as either cognitively stable or with accelerated cognitive decline based on their average performance in five neuropsychological tests (i.e., the Mini-Mental State Examination, the Benton Visual Retention Test, the Isaac’s Set Test, and the Trail-Making Test part A and part B) across five follow-up visits across the 12-year study duration [35].

^b^ApoE genotype was considered dichotomously: presence of at least one ε4 allele.

^c^Blood pressure ≥140/90 mmHg or antihypertensive medication use.

^d^Glucose ≥7.2 mmol/L or antidiabetic medication use.

^e^Fasting plasma total cholesterol ≥6.2 mmol/L or lipid-lowering medication use.

^f^History of cardiovascular or cerebrovascular disease.

^g^Includes all antihypertensive drugs, calcium channel blockers, diuretics, beta-blockers, and drugs acting on the renin-angiotensin system.

^h^Includes all antidiabetic drugs except insulin.

^i^Includes all statins, fibrates, or bile acid sequestrants.

^j^Includes all psycholeptics and psychoanaleptics—antidepressants, psychostimulants, and nootropics.

^k^Practice and intensity of physical exercise was assessed using a physical activity questionnaire for the elderly [100]. Regular exercise was classified as doing sport regularly or having at least 1 h of leisure or household activity per day. Described in detail in [101].

^l^A Mediterranean diet score was generated by adding the scores for each food group considered to be part of the Mediterranean diet [102].

^m^Metabolites quantified within our sample. Only those with a significant association with HN are displayed. Full list of 853 metabolites available upon request.

^n^Lipids we previously identified as being associated with cognitive decline in our sample [42].

### Cell line and culture conditions

We used the immortalised human fetal hippocampal multipotent progenitor cell line *HPC0A07/03* (HPC; ReNeuron Ltd, UK) as described before [27]. HPCs were cultured in medium (constitution as previously described [27]) and grown on tissue culture flasks, incubated at 37 °C, with 5% CO_2_ and saturated humidity. Cells were routinely passaged at 80% confluency before being plated for experiments. For further detail, see Supplementary Materials and Fig. S1.

### In vitro neurogenesis assay

HPC0A07/03C cells were treated with participant serum during their proliferation and differentiation, as previously described [27]. As detailed in Fig. 1B, 1% serum was added to the cell culture during both proliferation (48 h) and differentiation (7 days) before being fixed in 4% paraformaldehyde and stained for proliferation and differentiation specific markers, respectively. For further detail, see Supplementary Materials.

### Immunocytochemistry

Cell count, progenitor cell integrity, proliferation, cell death and differentiation were visualised using 4′,6-diamidino-2-phenylindole (DAPI), Nestin and SRY-Box Transcription Factor 2 (SOX2), Ki67, cleaved caspase-3 (CC3), doublecortin (DCX), and microtubule-associated protein 2 (MAP2) using immunocytochemistry, as previously described [27, 44]. For further detail, see Supplementary Materials and Fig. S2.

### Image analysis

All immunostainings were quantified using the unbiased, semi-automated CellInsight NXT High Content Screening platform and Studio Cell Analysis Software (ThermoScientific), as previously described [27, 44]. For further detail, see Supplementary Materials.

### Neurite outgrowth

To quantify the neurite outgrowth and branching of neuronal cells after differentiation, automated neurite outgrowth analyses were performed. Images were acquired using the CellInsight (as above) and analysis was performed using the web-based Columbus Analysis System (Perkin Elmer) and the CSIRO Neurite Analysis 2 method, as previously described [45]. For protocol details, see Supplementary Materials.

All experiments, immunocytochemistry, and image analyses were performed by an experimenter blinded to depressive status. All experiments were carried out in technical triplicates.

### Statistical analysis

Data analyses were conducted using SPSS Statistics 26 and R software (version 3.6.3). Logistic and linear regression models were used to study the association between HN readouts and depressive symptomology, and nutrient, metabolomic, and lipidomic biomarkers, respectively. Additionally, mediation and moderation analyses were conducted using the PROCESS macro, as previously described [46], to (i) more fully explore the relationship between neurogenesis, diet and depressive symptomology, and (ii) determine (where relevant) how key risk factors (e.g., CD, hippocampal volume, stress and inflammation) may influence the relationship between neurogenesis and depressive symptomology. All models were primarily adjusted for age, gender, education, and CD status, and in case of association, further adjustment was performed by including baseline depression status and relevant potential confounders (Tables 1–2). False discovery rate correction was applied to account for multiple testing throughout and all models were bootstrapped enhanced to obtain robust estimates of standard errors. For further detail, see Supplementary Materials.

## Results

### Cohort characteristics

Table 1 details the characteristics of our sample. Specifically, participants were on average 76 years old at baseline, 66% were female and 29% had a secondary school education or higher.

Within our sample, 8% of participants reported depressive symptomology at baseline, which increased to 30% across the duration of the study. Of this 30%: 18% experienced symptoms at a single timepoint (2% at baseline only), while the remaining 12% repeatedly reported symptoms (5% including baseline).

To start, we determined the association between individual HN markers and depressive symptomology reported at any timepoint, irrespective of chronicity. Only altered apoptosis during proliferation (i.e., %CC3- and %Ki67/CC3-positive cells; highlighted in blue in Table 1) and neuronal differentiation (i.e., %MAP2-positive cells and associated morphology; all highlighted in purple in Table 1) were significantly associated with depressive symptoms.

### A drive towards hippocampal stem cell differentiation may be associated with depressive symptomology

**Fig. 2:**
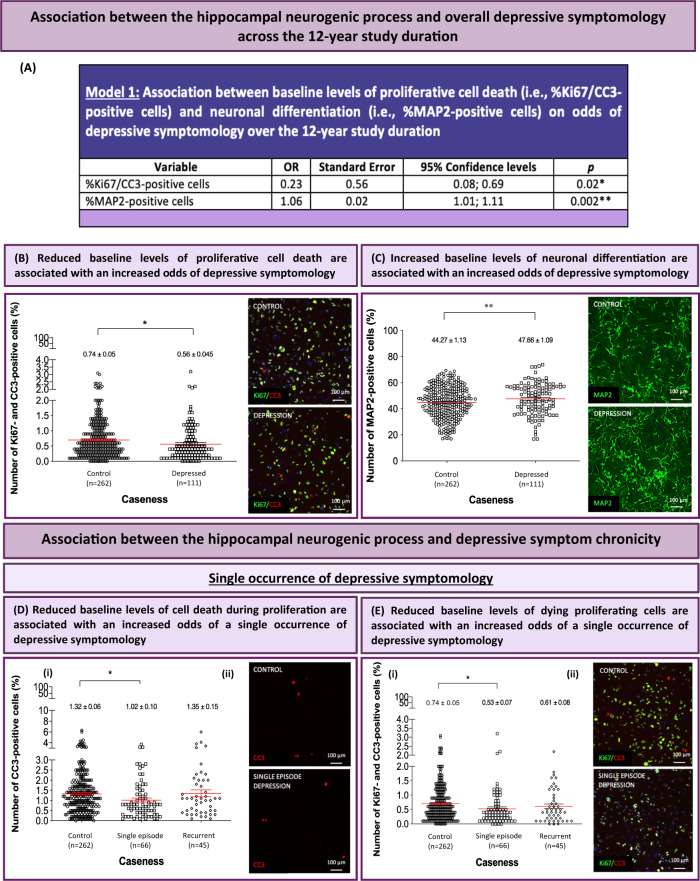

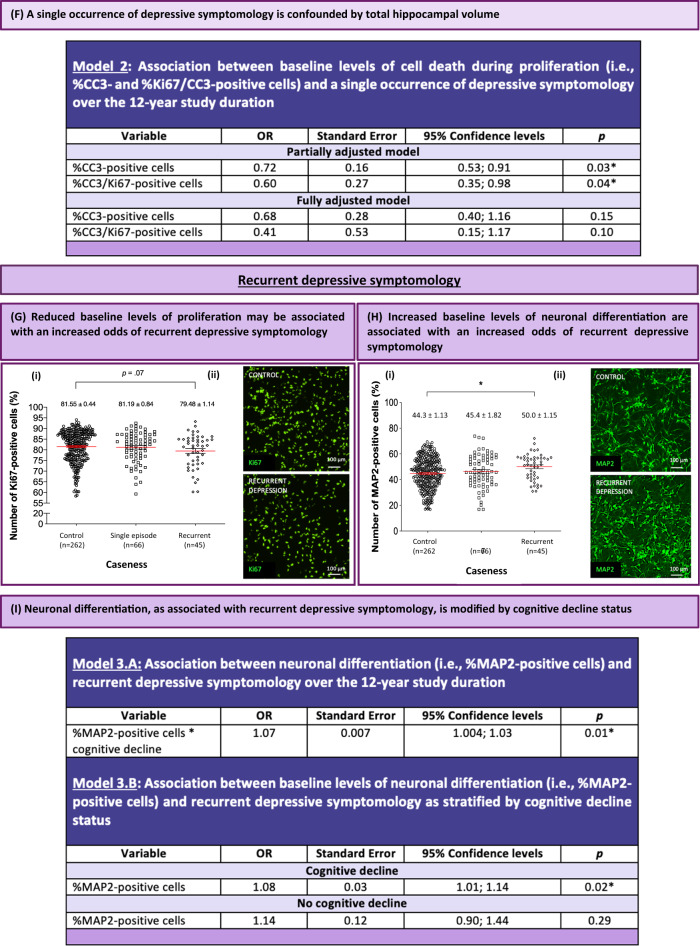
Relationship between the hippocampal neurogenic process and depressive symptomology and chronicity across the 12-year study duration. **A** Model 1: Association between baseline proliferative cell death (i.e., %Ki67/CC3) and neuronal differentiation (i.e., %MAP2) and depressive symptomology using logistic regression. Reduced baseline levels of %Ki67/CC3-positive cells (OR 0.23 [95% CI; 0.08 to 0.69]; *p* = 0.02) and increased %MAP2-positive cells (OR 1.06 [95% CI; 1.01 to 1.11]; *p* = 0.002) were both independently associated with depressive symptomology across the 12-year study period in a fully adjusted model. Model adjusted for age, gender, education, cognitive decline status, baseline depression, plasma glucose levels and total hippocampal volume. *p < 0.05; **p < 0.01. **B** (i) Baseline levels of %Ki67/CC3-positive cells stratified by caseness for depressive symptomology. Cases, i.e., those scoring positive for depressive symptomology at least once across the 12-year study (including at baseline), had significantly reduced levels of baseline levels of %Ki67/CC3-positive cells (M = 0.74 (0.05) vs. M = 0.56 (0.05)). Cellular readout expressed as a percentage relative to neural cell number. Cell line: *HPC0A07/03*; Passage number: P15-21; Technical replicates: *n* = 3; Data represents mean ± SEM. *p < 0.05. (ii) Representative immunostaining demonstrating %Ki67/CC3-positive cells for representative case and control. Images taken at x10 objective; scale bar represents 100 µm. **C** (i) Baseline levels of %MAP2-positive cells stratified by caseness for depressive symptomology. Cases had significantly increased levels of baseline %MAP2-positive cells (M = 44.3 (1.13) vs. M = 47.7 (1.09)). Cellular readout expressed as a percentage relative to neural cell number. Cell line: *HPC0A07/03*; Passage number: P15-21; Technical replicates: *n* = 3; Data represents mean ± SEM. **p < 0.01. (ii) Representative immunostaining demonstrating %MAP2-positive cells for representative case and control. Images taken at x10 objective; scale bar represents 100 µm. **D** (i) Baseline levels of %CC3-positive cells stratified by caseness for a single occurrence of depressive symptomology. Cases, i.e., those scoring positive for depressive symptomology only once across the 12-year study duration (including baseline), had significantly reduced levels of baseline levels of %CC3-positive cells (M = 1.02 (0.10) vs. M = 1.32 (0.06)). Cellular readout expressed as a percentage relative to neural cell number. Cell line: *HPC0A07/03*; Passage number: P15-21; Technical replicates: *n* = 3; Data represents mean ± SEM. *p < 0.05. (ii) Representative immunostaining demonstrating %CC3-positive cells during proliferation for representative case and control. Images taken at x10 objective; scale bar represents 100 µm. **E** (i) Baseline levels of %Ki67/CC3-positive cells stratified by caseness for a single occurrence of depressive symptomology. Cases had significantly reduced levels of baseline levels of %Ki67/CC3-positive cells (M = 0.53 (0.07) vs. M = 0.70 (0.03)). Cellular readout expressed as a percentage relative to neural cell number. Cell line: *HPC0A07/03*; Passage number: P15-21; Technical replicates: *n* = 3; Data represents mean ± SEM. *p < 0.05. (ii) Representative immunostaining demonstrating %Ki67/CC3-positive cells during proliferation for representative case and control. Images taken at x10 objective; scale bar represents 100 µm. **F** Important risk factors for a single occurrence of depressive symptomology: Model 2: Effect of baseline overall cell death and proliferative cell death on a single occurrence of depressive symptomology using logistic regression. Baseline levels of %CC3-positive cells during proliferation (OR 0.72 [95% CI; 0.53 to 0.91]; *p* = 0.03) and %Ki67/CC3-positive cells (OR 0.60 [95% CI; 0.35 to 0.98]; *p* = 0.04) were both associated with a single occurrence of depressive symptomology across the study period in a partially adjusted model (controlling for age, gender, education, and cognitive decline). However, these neurogenesis readouts were no longer significant in a fully adjusted model (controlling for age, gender, education, cognitive decline status, baseline depression, hypercholesterolemia, antecedents of cardiovascular disease, total hippocampal volume, cortisol levels and vitamin D supplementation) and were confounded by hippocampal volume (OR 0.98 [95% CI; 0.98 to 0.99]; *p* = 0.04). *p < 0.05. **G** (i) Baseline levels of %Ki67-positive cells (during proliferation) stratified by caseness for recurrent depressive symptomology. Cases, i.e., those scoring positive for depressive symptomology repeatedly across the 12-year study period, had a trend for reduced levels of baseline levels of %Ki67-positive cells during differentiation (M = 79.5 (1.14) vs. M = 81.6 (0.44)). Cellular readout expressed as a percentage relative to neural cell number. Cell line: *HPC0A07/03*; Passage number: P15-21; Technical replicates: *n* = 3; Data represents mean ± SEM. *p < 0.05. (ii) Representative immunostaining demonstrating %Ki67-positive cells during proliferation for representative case and control. Images taken at x10 objective; scale bar represents 100 µm. **H** (i) Baseline levels of %MAP2-positive cells stratified by caseness for recurrent depressive symptomology. Cases had significantly increased levels of baseline %MAP2-positive cells (M = 50.3 (1.48) vs. M = 44.8 (0.70)) relative to controls. Cellular readout expressed as a percentage relative to neural cell number. Cell line: *HPC0A07/03*; Passage number: P15-21; Technical replicates: *n* = 3; Data represents mean ± SEM. *p < 0.05. (ii) Representative immunostaining demonstrating %MAP2-positive cells for representative case and control. Images taken at x10 objective; scale bar represents 100 µm. **I** %MAP2-positive cell density, as associated with recurrent depressive symptomology, was modified by cognitive decline. Model 3.A: There was a significant interaction between %MAP2 and cognitive decline in participants with recurrent depressive symptomology (OR 1.07 [95% CI; 1.004 to 1.03]; *p* = 0.01). Model3.B: Further analyses revealed that %MAP2 was only associated with recurrent depressive symptomology in participants that also had cognitive decline (OR 1.08 [95% CI; 1.01 to 1.14]; *p* = 0.02). Models 3.A-B adjusted for age, gender, education, baseline depression, glucose levels, IL6 plasma levels, and regular physical exercise. *p < 0.05. CC3 cleaved caspase 3, MAP2 microtubule-associated protein 2, IL interleukin, OR odds ratio, P passage, M mean.

As depicted in Fig. 2A–C, decreased baseline levels of %Ki67/CC3-positive cells (i.e., dying proliferating cells; *p* = 0.02) and increased baseline levels of %MAP2-positive cells (i.e., young neurons; *p* = 0.002) were significantly associated with depressive symptomology within our sample across the 12-year period.

Having found that a decrease in the apoptosis of proliferating cells and an increase in neuronal differentiation was associated with depressive symptomology overall, we next sought to determine whether these HN outcomes would change depending on symptom chronicity.

### Reduced proliferative cell death is associated with a single occurrence of depressive symptomology but is confounded by hippocampal volume

**Fig. 3:**
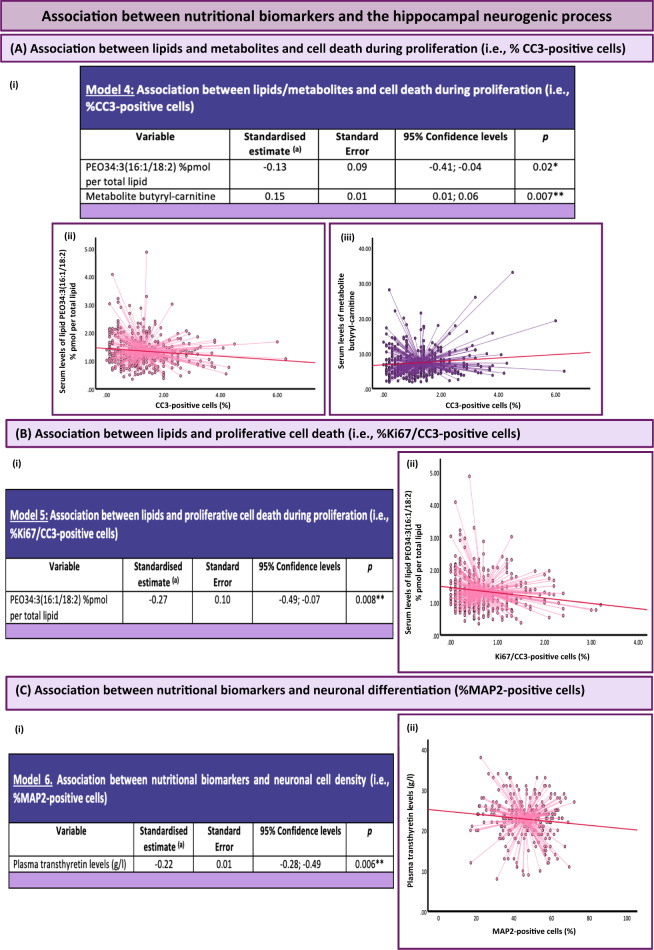

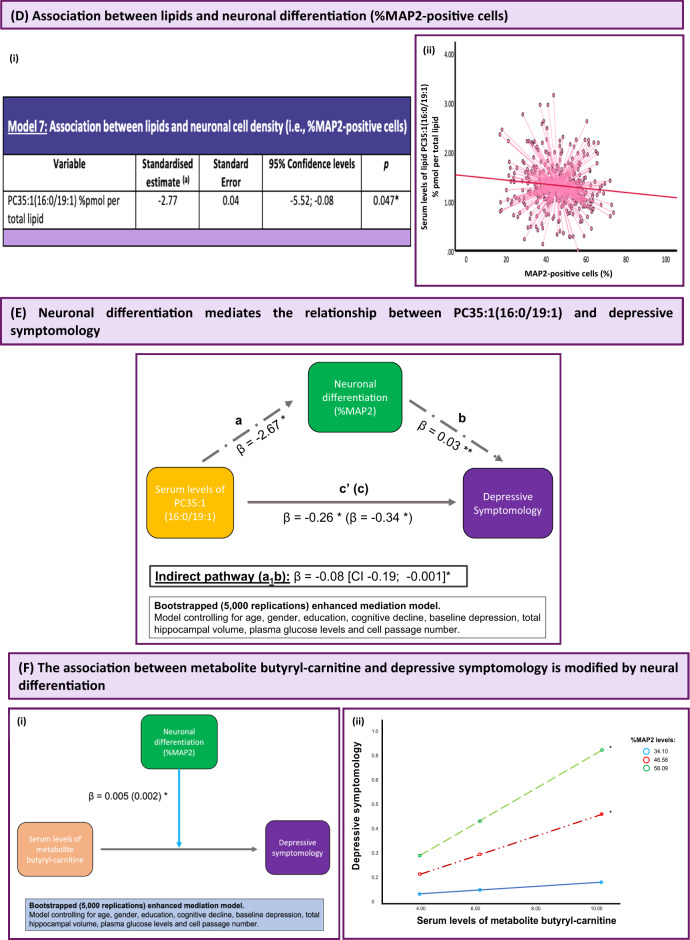
Relationship between the hippocampal neurogenic process, nutritional biomarkers, metabolites and lipids, and depressive symptomology. **A** Association between lipid and metabolite levels and cell death during proliferation. (**i**) Model 4: Association between lipid and metabolite levels and baseline levels of cell death during proliferation using linear regression. Baseline serum levels of lipid PEO34:3(16:1/18:2) (*ß* = −0.13 [95% CI; −0.41 to −0.04] 0.09; *p* = 0.02), and baseline serum levels of metabolite butyrylcarnitine (*ß* = 0.15 [95% CI; 0.01 to 0.06] 0.01; *p* = 0.007) were both associated with baseline %CC3-positive cell levels during proliferation in a fully adjusted model. Model adjusted for age, gender, education, cognitive decline status, baseline depression. (a) Increments are the estimates expressed as a 1-standard deviation increase. *p < 0.05; **p < 0.01. Scatterplot showing (ii) negative relationship between baseline serum levels of lipid PEO34:3(16:1/18:2) and %CC3-positive cells during proliferation at baseline (pink), and (iii) positive relationship between baseline serum levels of metabolite butyrylcarnitine and %CC3-positive cells during proliferation at baseline (purple). **B** Association between lipid levels and proliferative cell death. (i) Model 5: Association between lipid levels and baseline levels of proliferative cell death using linear regression. Baseline serum levels of lipid PEO34:3(16:1/18:2) (*ß* = −0.27 [95% CI; −0.49 to −0.07] 0.10; *p* = 0.008) were associated with baseline %Ki67/CC3-positive cell levels during proliferation in a fully adjusted model. Model adjusted for age, gender, education, cognitive decline status, baseline depression, hippocampal volume, and Mediterranean diet score. (a) Increments are the estimates expressed as a 1-standard deviation increase. **p < 0.01. Scatterplot showing (ii) negative relationship between baseline serum levels of lipid PEO34:3(16:1/18:2) and %Ki67/CC3-positive cells during proliferation at baseline (pink). **C** Association between nutritional biomarker levels and differentiation. (i) Model 6: Association between nutritional biomarker levels and baseline levels of neuronal cell differentiation using linear regression. Reduced plasma levels of transthyretin (*ß* = −0.22 [95% CI; −0.28 to −0.49] 0.01; *p* = 0.006) were associated with increased baseline %MAP2-positive cell levels in a fully adjusted model. Model adjusted for age, gender, education, cognitive decline status, baseline depression, zeaxanthin levels, arachidonic acid levels, retinol levels and cell passage number. (a) Increments are the estimates expressed as a 1-standard deviation increase. **p < 0.01. (ii) Scatterplot showing negative relationship between baseline plasma transthyretin levels and %MAP2-positive cells at baseline (pink). **D** Association between lipid levels and differentiation. (i) Model 7: Association between lipid levels and baseline levels of neuronal cell differentiation using linear regression. Reduced serum levels of lipid PC35:1(16:0/19:1) (*ß* = −2.77 [95% CI; −5.52 to −0.08] 0.01; *p* = 0.047) were associated with increased baseline %MAP2-positive cell levels in a fully adjusted model. Model adjusted for age, gender, education, cognitive decline status, baseline depression, PC32:2(14:0/18:2) levels, PC34:3(16:1/18:2) levels and cell passage number. (a) Increments are the estimates expressed as a 1-standard deviation increase. *p < 0.05. (ii) Scatterplot showing negative relationship between baseline serum levels of lipid PC35:1(16:0/19:1) and %MAP2-positive cells at baseline (pink). **E** There was a significant indirect effect of baseline serum levels of lipid PC35:1(16:0/19:1) on depressive symptomology through baseline %MAP2-positive cell levels (*ab* = −0.08 [−0.19; −0.001]). The mediator (i.e., %MAP2-positive levels) accounted for 24% of the total effect (*P*_*M*_ = 0.24). **F** The association between metabolite butyrylcarnitine and depressive symptomology was modified by neuronal differentiation. (i) There was a significant interaction between serum levels of metabolite butyrylcarnitine and %MAP2-positive cell levels (*b* = 0.005; SE = 0.002; *p* = 0.04). (ii) Interaction plot revealing that the positive association between serum levels of metabolite butyrylcarnitine and depressive symptomology was only significant for participants that had %MAP2 levels greater than 47% in our sample (*p* = 0.02). CC3 cleaved caspase 3, MAP2 microtubule-associated protein 2.

As shown in Fig. 2D, participants classified as experiencing depressive symptoms at a single timepoint across the study had significantly reduced baseline levels of %CC3-positive cells during proliferation (i.e., overall cell death; *p* = 0.03), in addition to a specific reduction in %Ki67/CC3 (i.e., dying proliferating cells; *p* = 0.04; Fig. 2E). However, when controlling for hippocampal volume, these associations no longer held (Fig. 3F).

### Increased neuronal differentiation and impaired neuronal cell morphology is associated with recurrent depressive symptomology but is modified by cognitive decline

Unlike a single occurrence of depressive symptomology, we found that increased baseline levels of %MAP2-positive cells were significantly associated with experiencing recurrent depressive symptoms across the course of the study (*p* = 0.02; Fig. 2H). Additionally, although not reaching statistical significance, we also observed a decrease in the level of %Ki67-positive cells (i.e., overall proliferation) for participants with recurrent depressive symptoms (*p* = 0.07; Fig. 2G). Moreover, although participants with recurrent depressive symptomology had more %MAP2-positive cells, morphological analyses revealed a significantly impaired morphology, such that these MAP2-positive cells had a reduced total neurite length (*p* = 0.003), fewer number of neurites (*p* = 0.005) and a less complex degree of neurite branching (*p* = 0.006) (Table 1).

Interestingly, in our fully adjusted model, we found a significant interaction between %MAP2 and CD amongst those with recurrent depressive symptoms (*p* = 0.01), revealing that these changes in %MAP2 were only associated with recurrent depressive symptomology in those also diagnosed with CD (*p* = 0.02; Fig. 2I).

Thus far, we have shown that a potential drive towards hippocampal stem cell differentiation may be associated with late-life depressive symptomology, and that CD can modify the association of %MAP2 in participants experiencing recurrent depressive symptoms. No differences in any other individual neurogenesis readout, i.e., %Nestin-, %SOX2-, %CC3-(during differentiation) or %DCX-positive cells, were observed between groups (Table 1). Moreover, we found no difference in the neurite morphology of %DCX- and %MAP2-positive cells between depressive symptomology (overall), or a single depressive episode, and controls (Table 1). Additionally, principal component analyses revealed no differences in the overall neurogenic profiles between groups (Supplementary Materials).

Next, as detailed in Table 2, we assessed whether nutritional status could modulate these changes in %CC3 (prol), %Ki67/CC3 and %MAP2.

### Neuronal differentiation mediates the relationship between PC35:1(16:0/19:1) and depressive symptomology, while the association between metabolite butyrylcarnitine and depressive symptomology is modified by neural differentiation

As shown in Fig. 3, reduced serum levels of lipid PE034:3(16:1/18:2) (*p* = 0.02) and increased serum levels of metabolite butyrylcarnitine (*p* = 0.007) were both independently associated with increased baseline levels of %CC3-positive cells during proliferation in a fully adjusted model (Fig. 3A). Additionally, we also found a negative association between serum levels of lipid PE034:3(16:1/18:2) and baseline %Ki67/CC3 (*p* = 0.008; Fig. 3B). Moreover, reduced plasma levels of transthyretin (*p* = 0.006; Fig. 3C), and serum levels of glycerophospholipid, phosphatidylcholine [PC]35:1(16:0/19:1) (*p* = 0.047; Fig. 3D) were both independently associated with increased %MAP2.

While we found that several nutritional measures were associated with HN in our sample, only metabolite butyrylcarnitine and lipid PC35:1(16:0/19:1) were also associated with depressive symptomology. Specifically, we found a significant positive association between butyrylcarnitine levels (*p* = 0.049), and a negative association between PC35:1(16:0/19:1) levels (*p* = 0.045), and depressive symptoms.

To understand the relationship(s) more fully between this metabolite and phospholipid, our HN outcomes, and depressive symptomology, mediation analyses were subsequently performed. As depicted in Fig. 3E, we found a significant indirect effect of  serum PC35:1(16:0/19:1) levels (*ab* = −0.08 [−0.19; −0.01]; *p* = 0.04) on depressive symptomology as mediated through %MAP2 levels.

While we found no significant indirect effect of butyrylcarnitine levels on depressive symptomology as mediated through %CC3, we did however find a significant interaction between %MAP2 and this metabolite (*p* = 0.04). Therefore, we performed a simple moderation analysis and found that those with higher levels of %MAP2 (i.e., <46%, *p* = 0.03; <58%, *p* = 0.01) and increased levels of butyrylcarnitine were more likely to have depressive symptoms (*p* = 0.005; Fig. 3F).

## Discussion

Using serum samples from a longitudinal, population-based ageing cohort, we provide evidence to support that blood-borne factors, via the systemic milieu of participants, influence the fate of hippocampal progenitor cells in vitro, notably in association with late-life depressive symptomology. We demonstrate that both reduced baseline levels of apoptotic, proliferating cells (i.e., %Ki67/CC3-positive cells) and increased baseline differentiation (i.e., %MAP2-positive cells) are independently associated with the occurrence of depressive symptomology across a 12-year period in later life. Moreover, these neurogenesis outcomes appear to be context-specific regarding the chronicity and recurrence of depressive symptoms. For example, reduced proliferative cell apoptosis was uniquely associated with experiencing depressive symptomology at a single timepoint, whereas increased neuronal differentiation was a hallmark of recurrent symptomology within our cohort. Furthermore, we demonstrated that these alterations in neurogenesis were modulated by metabolomic and lipidomic biomarkers, i.e., butyrylcarnitine and PC35:1(16:0/19:1), and that diet could thus play an important role in regulating the neurogenic process in humans.

The hippocampus has been implicated in learning and memory [47, 48], stress responsivity [49, 50] and emotional regulation [51] factors all associated with, and often altered in, MDD [52, 53]. The hippocampus is also unique in that it contains one of the niches within the human brain where neurogenesis occurs [54]. Our present in vitro data suggest that depressive symptoms are not only associated with reduced hippocampal volume—one of the most replicated neuroimaging findings in MDD research [55], but also that HN may play a key role in the pathogenesis and/or progression of depression.

In the context of our work, we found a specific decrease in the number of dying proliferative cells during the earlier, proliferation phase of the neurogenic process, without a concomitant decrease in overall cell death. We also observed a subsequent increase in the number of neurons during differentiation, which may be a ‘knock-on’ effect of the changes occurring in the earlier stages of the neurogenic process. Taken together, this suggests that neuronal cells might be pushed towards differentiation in late-life depression, which, importantly, is not attributable to antidepressant medication here, as previously described [56–58]. Interestingly, an increase in peripheral levels of MAP2 in bipolar depression has recently been reported as well [59], while one post-mortem study also showed an increase in pyramidal neuronal density in the CA1 in chronic MDD [60]. However, it is notable that the literature predominately reports a reduction in all neurogenesis-associated readouts in depression [61], although, these findings primarily stem from end-stage tissue samples from young-to-middle aged MDD patients; therefore, it is difficult to extrapolate these results to that of our own.

Our most exciting finding is that despite our unique sample and study, with prominent differences in time (12-year follow-up), approach and measures (cellular changes vs. depressive symptomatology), some specificity persisted nevertheless between our HN outcomes and the chronicity and/or recurrent nature of depressive symptomology. For example, the observed drive towards neuronal differentiation was unique to experiencing recurrent depressive symptoms within our cohort. Moreover, although we observed an increase in MAP2 neurons in our in vitro assay from participants with recurrent symptoms, the morphology of these neurons was significantly impaired; they had fewer, shorter neurites and a less complex branching pattern—a finding that has previously been reported in stress models [62, 63], in older individuals with psychological distress [64], and in the context of late-life depression [65]. Furthermore, several clinical studies have reported synaptic dysfunction in MDD, albeit in the prefrontal cortex, which may be one functional biological consequence of these observed morphological changes [66], of which MAP2 plays a key role [67]. However, whether the increased number of differentiating cells is a causal or adaptive response to these morphological impairments requires further substantiation.

One of the potentially wider functional implications of the changes in HN observed for those with recurrent depressive symptomology could relate to cognitive capacity [68]. Indeed, while neurogenesis was associated with recurrent depressive symptoms in our sample, this was only found in a subset of these participants, i.e., those that subsequently developed CD, highlighting the complex relationship between CD and depression particularly in later life. Our data suggest that either recurrent depressive symptoms could here represent a consequence, or concomitant event, of CD (and altered HN), or that altered HN could potentially modulate cognitive reserve, or exacerbate cognitive impairment in, those already suffering with recurrent depressive symptoms. Pertinently, an association between altered HN and CD has consistently been reported in rodent models [69], in addition to the recognised overlap between depression, CD and dementia for which neurogenesis may be an important mediator [61].

In the context of a single episode of depressive symptomology, although we observed a negative association with proliferative cell death, this was confounded by hippocampal volume. More specifically, reduced hippocampal volume increased the risk of a single depressive episode—a finding consistently reported in the literature [55]. Interestingly, we also observed a positive relationship between total hippocampal volume and this HN-associated outcome, potentially representing a compensatory response to loss of volume. However, mediation analyses did not support that the relationship between hippocampal volume and a single depressive episode was modulated by reduced proliferative cell death in our sample, although we are mindful that insufficient power upon sample stratification may have limited these analyses [70]. Future work should seek to explore the relationship more fully given that our understanding of the molecular and cellular processes contributing to hippocampal volume loss (and the role that HN plays) are not well-established [71, 72] and that this may represent an important predictive biomarker for depression and its associated recovery [73].

Given that the strongest risk factors for late-life depression are related to environmental and/or sociodemographic characteristics [74], it is unsurprising that in the context of a single, possibly, acute depressive episode that demographic factors such as education (which attributed most to the outcome) would be more relevant. Alterations in neurogenesis, in the context of depression, may only be relevant under more chronic forms of exposure, and/or, more importantly, in the presence of other key risk factors for depression – as we have demonstrated in the context of recurrent depressive symptomology. Meaningfully, we did find a negative relationship between cortisol levels (a biomarker for depression [75]) and a single episode of depressive symptomology, although this does contradict the literature predominately supporting a positive association [76]. However, it is noteworthy that in community-dwelling older adults, both hypo- and hypercortisolism have been associated with increased depressive symptomology and that hypocortisolism was present only in those with more health problems [77]. In our cohort we observed a similar outcome: those with a single bout of depressive symptomology also presented poorer health outcomes (i.e., increased hypercholesterolemia and antecedents of cardiovascular disease, Table 1). Poorer health status may therefore represent an additional burden in this subgroup, possibly leading to chronic stress and a blunted stress response.

Based on our findings that specific HN markers are associated with depression, we next sought to determine whether nutritional, metabolomic and/or lipidomic biomarkers could be modulating these outcomes, given the association between diet and neurogenesis [16, 17]. Interestingly, not only did we find that both metabolite butyrylcarnitine (a member of the acylcarnitines) and glycerophospholipid, PC35:1(16:0/19:1), were associated with depressive symptomology – findings that have previously been reported in clinical populations [78, 79] but that they also specifically modulated neuronal differentiation within our sample. For example, we show that reduced levels of PC35:1(16:0/19:1) were associated with increased differentiation, which in turn increased the risk of late-life depressive symptoms. Moreover, we find that the positive association between butyrylcarnitine levels and depressive symptomology is modified by differentiation, such that those individuals with higher levels of both butyrylcarnitine and neuronal differentiation are at the greatest risk.

Importantly, PCs are major components of the cell membrane, are involved in cell signalling and metabolism, and play a critical role in neurotransmission and synaptic plasticity [80, 81], while acytylcarnitines are required for β-oxidation and tricarboxylic acid cycle activity [81, 82]. Indeed, several studies demonstrate that the transition from neural stem cell to a neuronal lineage is accompanied by increased mitochondrial biogenesis, and a downregulation of glycolysis and fatty acid oxidation [81]. Thus, β-oxidation and lipogenesis play a critical role in neurogenesis and the dysregulation of these metabolic pathways could significantly impair the process. Our findings potentially support that there may be a metabolic dysregulation of neurodifferentiation in the context of late-life depression, given that we observe alterations in both butyrylcarnitine and PC35:1(16:0/19:1). Furthermore, these endogenously derived factors are closely related to diet [83, 84]. For example, the precursor of butyrylcarnitine is butyric acid, a short chain fatty acid whose main source is the microbial fermentation of dietary fibres in the gut [83]. Therefore, dietary modification in later life could represent a potential therapeutic target for depression.

Additionally, we also found that plasma levels of transthyretin – a biomarker for malnutrition [85] was negatively associated with neuronal differentiation. Malnutrition has consistently been associated with depression [86, 87], cognitive decline and/or dementia [88, 89], and plays an important role in altering brain plasticity, particularly during early life [90]. Interestingly, malnutrition is associated with increased apoptosis and immune system activation [91, 92], which might represent the mechanism of action by which overall nutrient deficiency alters neurogenesis. Moreover, we found a negative association between cell death during proliferation and (PE0)34:3(16:1/18:2) – an ether phospholipid that forms a key component of the lipid membrane and lipid rafts [93]. Ether phospholipids, together with cell death, not only plays a critical role in neurotransmission and synaptic plasticity [80], but has also been implicated in the pathogenesis of depression [79]. However, the precise mechanisms involved in how these nutritional factors influence HN outcomes remain to be elucidated, falling outside the scope of this study. Moreover, we are mindful that in our sample, neither transthyretin or PE034:3(16:1/18:2) levels were significantly associated with depressive symptomology and more work is needed to substantiation these findings in the context of depression.

Of note, consistent with previous research [94, 95], we did find a positive association between inflammation (i.e., plasma levels of IL6) and recurrent depressive symptomology, which could potentially represent another mechanism of action for this particular subgroup. Given that cytokines, like IL6, are important regulators of HN [96], it is possible that increased neuronal differentiation (as a potential consequence of altered neuronal apoptosis) may be associated with immune system dysfunction in chronic instances of late-life depression. However, we found no statistical evidence to support that the relationship between neuronal differentiation and recurrent depression was modulated by IL6 levels in our sample.

The strength of our work lies in the use of a well-characterised prospective cohort to evaluate the impact of nutrition (including metabolomics and lipidomics) on in vitro neurogenesis measures in the trajectory of late-life depressive symptomology. However, our study also has limitations. First, our HN measures are only proxy measures of in vitro neurogenesis; therefore, might not mirror those in vivo. It is also unclear whether these observed HN changes are causal or adaptive, given that this was a cross-sectional evaluation of neurogenesis. Moreover, we recognise that our assay does not reconstitute the neurogenic niche in its entirety, and future work should expand the model to include other important HN regulators such as microglia [97], particularly given the important association between depression and (neuro)inflammation, which could ultimately affect the outcomes presented herein [95, 98]. Finally, it would be profitable for future research to also extend the assay’s duration to monitor synaptic formation and plasticity (considering our morphological findings), and to adopt a longitudinal approach and compare HN markers and overall neurogenic profiles at multiple time points to understand the impact of the hippocampal neurogenic process more fully across the trajectory of late-life depression.

In summary, given that there are currently no methods of quantifying HN in living humans, our assay presents a powerful tool to better understand the relationship between diet, neurogenesis, and depression. More work is now needed to more fully understand how these metabolites/lipids can modulate HN in the context of depression, but we have shown that HN, modulated by butyrylcarnitine and PC35:1(16:0/19:1), is associated with late-life depressive symptomology, and that we can distinguish between HN-associated outcomes and symptom chronicity. Our work is particularly important given that current treatment options for depression are often limited [99], and, thus, diet may represent a promising way via which the burden imposed by this debilitating condition could be alleviated.

## Supplementary information

SUPPLEMENTARY MATERIAL
