# Supplementary material for: Impaired hippocampal neurogenesis in vitro is modulated by dietary-related endogenous factors and associated with depression in a longitudinal ageing cohort study
Source: Mol Psychiatry. 2022 Jul 7;27(8):3425–40. doi: 10.1038/s41380-022-01644-1 (PMC7613865; doi:10.1038/s41380-022-01644-1)
Supplement: Supplementary file 1 — SUPPLEMENTARY MATERIAL [file 41380_2022_1644_MOESM1_ESM.pdf]

SUPPLEMENTARY MATERIAL

Impaired hippocampal neurogenesis *in vitro* is modulated by dietary-related endogenous factors and associated with depression in a longitudinal ageing cohort study

Andrea Du Preez[1], Sophie Lefèvre-Arbogast[2], Raúl González-Domínguez[3,4], Vikki Houghton[1], Chiara de Lucia[1], Dorrain Y Low[5], Catherine Helmer[2], Catherine Féart[2], Cécile Delcourt[2], Cécile Proust-Lima[2], Mercè Pallàs[6], Alex Sánchez-Pla[3,4], Mireia Urpi-Sardà[3,4], Silvie R. Ruigrok[7], Barbara Altendorfer[8], Ludwig Aigner[8], Paul J Lucassen[7], Aniko Korosi[7], Claudine Manach[5], Cristina Andres-Lacueva[3,4], Cécilia Samieri[2], Sandrine Thuret[1, 9]

[1] Department of Basic and Clinical Neuroscience, Maurice Wohl Clinical Neuroscience Institute, Institute of Psychiatry, Psychology and Neuroscience, King's College London, London SE5 9NU, UK; [2] University of Bordeaux, Inserm, Bordeaux Population Health Research Center, UMR 1219, F-33000 Bordeaux, France; [3] Nutrition, Food Science and Gastronomy Department, Faculty of Pharmacy and Food Science, University of Barcelona, 08028 Barcelona, Spain; [4] CIBER Fragilidad y Envejecimiento Saludable (CIBERFES), Instituto de Salud Carlos III,0828, Barcelona; [5] Université Clermont Auvergne, INRA, UMR1019, Human Nutrition Unit, F-63000 Clermont Ferrand, France; [6] Pharmacology Section, Department of Pharmacology, Toxicology and Medicinal Chemistry, Faculty of Pharmacy and Food Sciences, and Institute of Neurosciences, University of Barcelona, Av. Joan XXIII, 27-31, E-08028, Barcelona, Spain; [7] Brain Plasticity Group, Swammerdam Institute for Life Sciences, Center for Neuroscience, University of Amsterdam, 1098 XH Amsterdam, The Netherlands; [8] Institute of Molecular Regenerative Medicine, Spinal Cord Injury and Tissue Regeneration Center Salzburg, Paracelsus Medical University, Salzburg 5020, Austria; [9] Department of Neurology, University Hospital Carl Gustav Carus, Technische Universität Dresden, 01307 Dresden, Germany.

Keywords: Adult hippocampal neurogenesis, butyrylcarnitine, phospholipids, depressive symptomology, symptom chronicity, ageing.

Correspondence to:
Prof. Sandrine Thuret
Email: Sandrine.1.thuret@kcl.ac.uk

CONTENT

Methods and materials

Results

METHODS AND MATERIALS

Cohort and study design

A case-control study on cognitive decline (CD) nested within the 3C-Bordeaux cohort (n=2,104) was constructed to investigate the relationships between variations in the serum metabolome, the neurogenic process and subsequent CD and depression. From this cohort, a total of 1,293 participants were free from CD at baseline and of these only 418 participants were retained for case-control sampling, having had at least one repeated cognitive evaluation over the 12-year follow-up and an available baseline ser2um sample. Given the focus of our work was on depression, of these 418 participants, 373 had data on depressive symptomology and were included in our analyses. Sample size was determined from power calculations based on previous work (1,2). Simulation studies suggested a power of 80% using a sample size of ~400 subjects.

The 3C study protocol was approved by the Consultative Committee for the Protection of Persons participating in Biomedical Research at Kremlin-Bicetre University Hospital (Paris, France). All participants provided written informed consent prior to participation.

3C-Bordeaux baseline measures

As previously described ((3–6), at baseline, face-to-face interviews were conducted to collect sociodemographic and lifestyle characteristics, medical information, cognitive testing, blood pressure, and anthropometric measurements from all participants. Additionally, fasting serum and plasma samples collected at baseline and stored at −80°C in a biobank. The plasma samples were used for the nutrient biomarkers (7–10) and the serum samples were used for the metabolomics (6), lipidomics (11) and *in vitro* cellular assays.

More generally the following variables were collected across the course of the study: brain related measures: cognitive function, mental health, neurological measures; functional rating: individual psychological health; anthropometric: blood pressure, height and weight;

physical: cardiovascular and diabetes; biological: immune markers, vitamin screening; APOE carrier status; brain imaging: magnetic resonance imaging (MRI); lifestyle: alcohol use, dietary habits, energy intake, physical activity and smoking; socioeconomic: education, income and finances, occupation and employment; nutrient: fatty acid biomarkers, carotenoid biomarkers and vitamin D and malnutrition biomarkers.

Assessment of depressive symptoms

Follow-up visits were performed every 2-3 years, across 12 years, during which extensive neuropsychological assessments were carried out by a trained psychologist. Depressive symptomatology at baseline and throughout the 12-year follow-up was evaluated using the validated Center for Epidemiologic Studies Depression (CES-D) scale (12), which is also a valid and reliable measure of depressive symptomatology in old age populations (13,14). The CES-D is a 20-item, self-report questionnaire designed to evaluate the frequency of depressive symptoms experienced over the past week. Each item is scored from 0 (rarely) to 3 (most of the time), with a total score ranging from 0 to 60 and increasing with the level of severity of depressive symptomatology. As previously validated in a French population, scores of ≥17 in men and of ≥23 in women were used as indicators of a clinically relevant level of depressive symptomatology (12,15,16). When the CES-D scale was not fully completed, the interviewers mentioned if it was because of severe depression. When more than four items out of 20 were missing because of severe depression as ascertained by the psychologist, the participant was considered as having high depressive symptoms. Caseness for depressive symptomology was not based on antidepressant use, e.g., participants with no depressive symptoms but taking psychoactive medication (including antidepressants) were classified as controls. However, only 30% of all participants, irrespective of symptomology, were taking medication during the study. Antidepressant use was however considered in all analyses.

Assessment of cognitive decline

Participants within our sample were classified as either cognitively stable or with accelerated cognitive decline (CD) based on an approach we developed and have previously published (6). Briefly, using linear mixed models, individual slopes of cognitive change for each participant

over the 12-year follow-up period were estimated. The primary outcome represented the change in a composite score of global cognition, which was defined as the average of Z-scores of five neuropsychological tests: (i) the Mini-Mental State Examination (assessing global cognitive performance)(17), (ii) the Benton Visual Retention Test (assessing visual working memory and attention)(18), (iii) the Isaac's Set Test (assessing verbal fluency)(19), (iv) the Trail-Making Test part A (assessing processing speed)(20), and (v) the Trail-Making Test part B (assessing executive functioning)(20), across the five follow-up visits. Models included an intercept, representing the level of composite cognitive score at baseline, a slope, indicating the annual change in scores over time, and both a random intercept and a random slope to account for inter-individual variability. Participants with the worst slopes of decline were classified as those with accelerated CD, whereas participants with a CD below median value (i.e., >median slope) were classified as cognitively stable.

Cell line

We used the human fetal hippocampal multipotent progenitor cell line *HPC0A07/03* (HPC; ReNeuron Ltd, Surrey, UK). Cells were sourced from 12-week-old female fetal tissue in accordance with UK and USA ethical and legal guidelines. As shown in **Figure S.1**, this cell line has been conditionally immortalised via infection with a retroviral vector pLNCX2, encoding the c-MycERTAM transgene construct. This transgene is solely activated by the synthetic steroid 4-hydroxytamoxifen (4-OHT) and as such, in the presence of 4-OHT, and growth factors: basic fibroblast growth factor (bFGF) and epidermal growth factor (EGF), cells can be continuously cultured without differentiating. Consequently, absence of 4-OHT, bFGF and EGF from the culture medium enables cells to differentiate into astrocytes, oligodendrocytes, and neurons. Based on previous work within the lab (21–23), one week of differentiation is sufficient to detect changes in the number of immature and mature neurons. Specifically, seven days of differentiation results in a cell population composed of 35% doublecortin (DCX)-positive immature neuroblasts, 25% microtubule associated protein 2 (MAP2)-positive neurons, 27% S100b-positive astrocytes and 2% O1-positive oligodendrocytes. The remaining population maintain a neural progenitor cell phenotype.

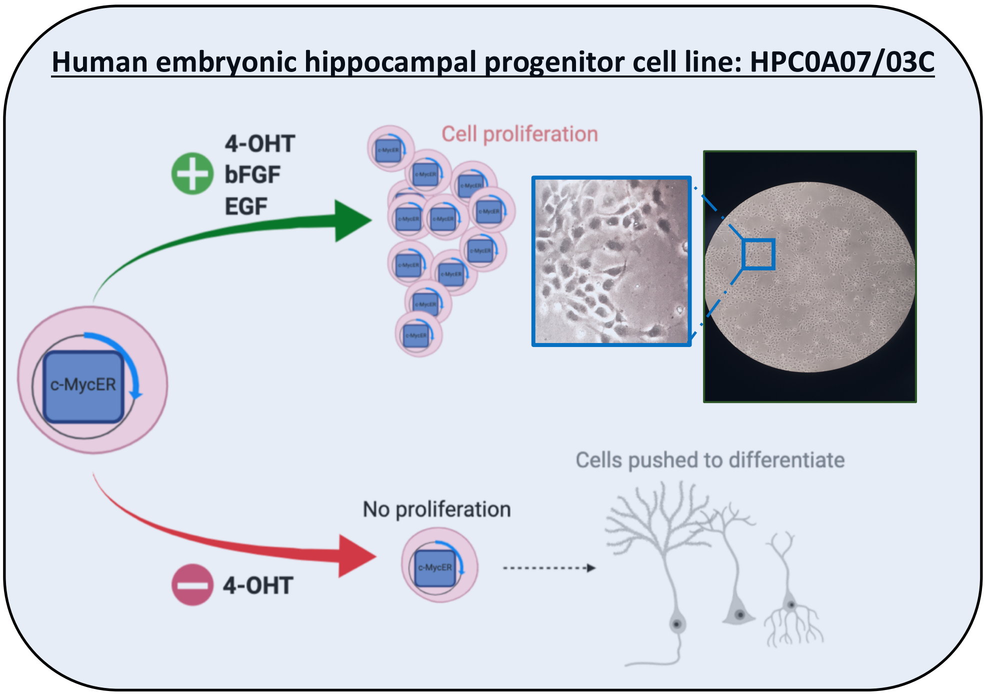

Figure S.1. **Schematic of transgene function in the *HPCOA07/03A* cell line by ReNeuron.**
The cell line contains the c-Myc oncogene under the control of the estrogen receptor (ER); c-MycER. In the presence of 4-hydroxytamoxifen (4-OHT; synthetic version of estrogen), c-MycER dimerizes and translocates to the nucleus. This allows promoter binding, which leads to the activation of downstream pathways leading to cell proliferation, provided growth factors: basic fibroblast growth factor (bFGF) and epidermal growth factor (EGF), are also present. In the absence of 4-OHT, the cells are unable to proliferate and begin differentiating. Image adapted from (24).

Cell culture

HPCs were cultured in reduced modified medium (RMM), namely Dulbecco's Modified Eagle's Media/F12 (DMEM: F12, Sigma), supplemented with 0.03% human albumin solution (Zenalb), 100 µg/mL human apo-transferrin, 16.2 µg/mL human putrescine diHCl, 5 µg/mL human recombinant insulin, 60 ng/mL progesterone, 2 mM L-glutamine and 40 ng/mL sodium selenite. For proliferation, the medium also included 10 ng/mL human basic fibroblast growth factor (bFGF), 20 ng/mL human epidermal growth factor (EGF) and 100nM 4-OHT. Cells were grown on tissue culture flasks (Nunclon, Denmark), incubated at 37°C, with 5% CO_2 and saturated humidity, and were routinely passaged at 80% confluency before being plated for experiments. All cultures were regularly checked for mycoplasma infection.

***In vitro* neurogenesis assay**

To measure how the systemic environment (i.e., serum) influences human hippocampal neurogenesis, HPC0A07/03C cells were treated with 1% participant serum (samples were from the same batch of serum used to derive the metabolomic and lipidomic data) in accordance with the *in vitro* assay we developed and optimised (2,25–27). Participant serum was added to the cell culture during both proliferation and differentiation. Briefly, 24 hours after seeding, cell medium (composition as aforementioned) was replaced with fresh medium containing 1% serum and 1:100 PenStrep (ThermoFisher Scientific) and was subsequently left to incubate for 72 hours before being fixed in 4% paraformaldehyde (PFA) stained for proliferation specific markers. To assess the systemic effects of serum on differentiation, after 48 hours of proliferation in the presence of 1% serum and 1:100 PenStrep, cells were washed and treated with another serum supplementation, this time in medium absent of 4-OHT, EGF and FGF to allow cells to spontaneously differentiate. Serum treated cells and were subsequently left to differentiate for a further 7 days before being fixed in 4% PFA and stained for differentiation specific markers.

Fasting serum samples were collected at baseline, stored at -80°C in a biobank until use and underwent one or two freeze/thaw cycles before performing the proliferation and differentiation experiments, respectively. Serum samples were never pooled for any experiment (i.e., represent an individual participant) and each serum sample had a technical triplicate for both proliferation and differentiation. In all assays, control conditions consisted of medium and 1:100 PenStrep (ThermoFisher Scientific) and all experiments were performed using cells of passage number ranging from 15 to 21. All *in vitro* cellular work was carried out by an experimenter blinded to the participant’s depressive status.

Immunocytochemistry

To analyse the fate and morphology of serum-treated HPCs, immunocytochemistry (ICC) was used as previously described (25,27–29). Briefly, PFA-fixed cells were incubated for 1h at room temperature in blocking solution, consisting of phosphate buffered saline (PBS) with sodium azide (NaN_3; 0.05% dilution), 5% normal donkey serum (D9963, Sigma) and 0.3%

Triton-X (93443, Sigma). Cells were then incubated in 30µl of primary antibodies: nestin and SOX2 for hippocampal progenitor integrity (Mouse anti-Nestin, 1:1000, Merck Millipore; Mouse anti-Nestin, 1:1000, Abcam); Ki67 for proliferation (Mouse anti-Ki67, 1:800, Cell Signalling Technology); cleaved caspase-3 for apoptosis during both proliferation and differentiation (CC3; rabbit anti-CC3, 1:500, Cell Signalling Technology); doublecortin for neuroblasts (DCX; rabbit anti-DCX, 1:500, Abcam) and microtubule-associated protein 2 for neurons (MAP2; mouse-anti MAP2, 1:500, Abcam) - all diluted in blocking solution overnight at 4°C. Cells were then washed twice with PBS, and incubated in blocking solution for 30 minutes at room temperature, before being further incubated for 2h, at room temperature, in the dark in 30µl of secondary antibodies diluted in blocking solution (Alexa 488 donkey anti-mouse, 1:500, Life Technologies, A-21202; Alexa 555 donkey anti-rabbit, 1:500, Life Technologies, A-31572). Cells were then washed twice with PBS and nuclei were stained with 50µl of 300µm 4′,6-diamidino-2-phenylindole solution diluted in PBS (DAPI; D9542-5mg, Sigma) for 5 minutes at room temperature. Finally, cells were washed twice more before being stored in 200µl PBS with NaN3 (0.05% dilution) at 4°C in the dark. For representative images of all immunostainings, see **Figure S.2.**

Image analysis

All immunostainings were quantified using the semi-automated CellInsight NXT High Content Screening (HCS) platform (ThermoScientific) and Studio Cell Analysis Software (Thermo Scientific) as previously described (25,27–29). This platform relies on light intensity thresholds, which identify DAPI (wavelength 386) or secondary antibody fluorescence (wavelengths 488 and 555). These thresholds, combined with other parameters based on cell size and shape, identify cells stained by each antibody in an unbiased way and enable semi-automated quantification of immunocytochemical stains.

Briefly, images were taken with a x10 objective that autofocuses using the DAPI stain. Exposure times for each channel were manually defined to ensure a good signal-to-background-noise ratio. All images underwent a background removal step for all channels using the software-recommended parameters for intensity-relevant measures. Individual cells were determined using the DAPI-positive nuclei, and smoothing, threshold, and

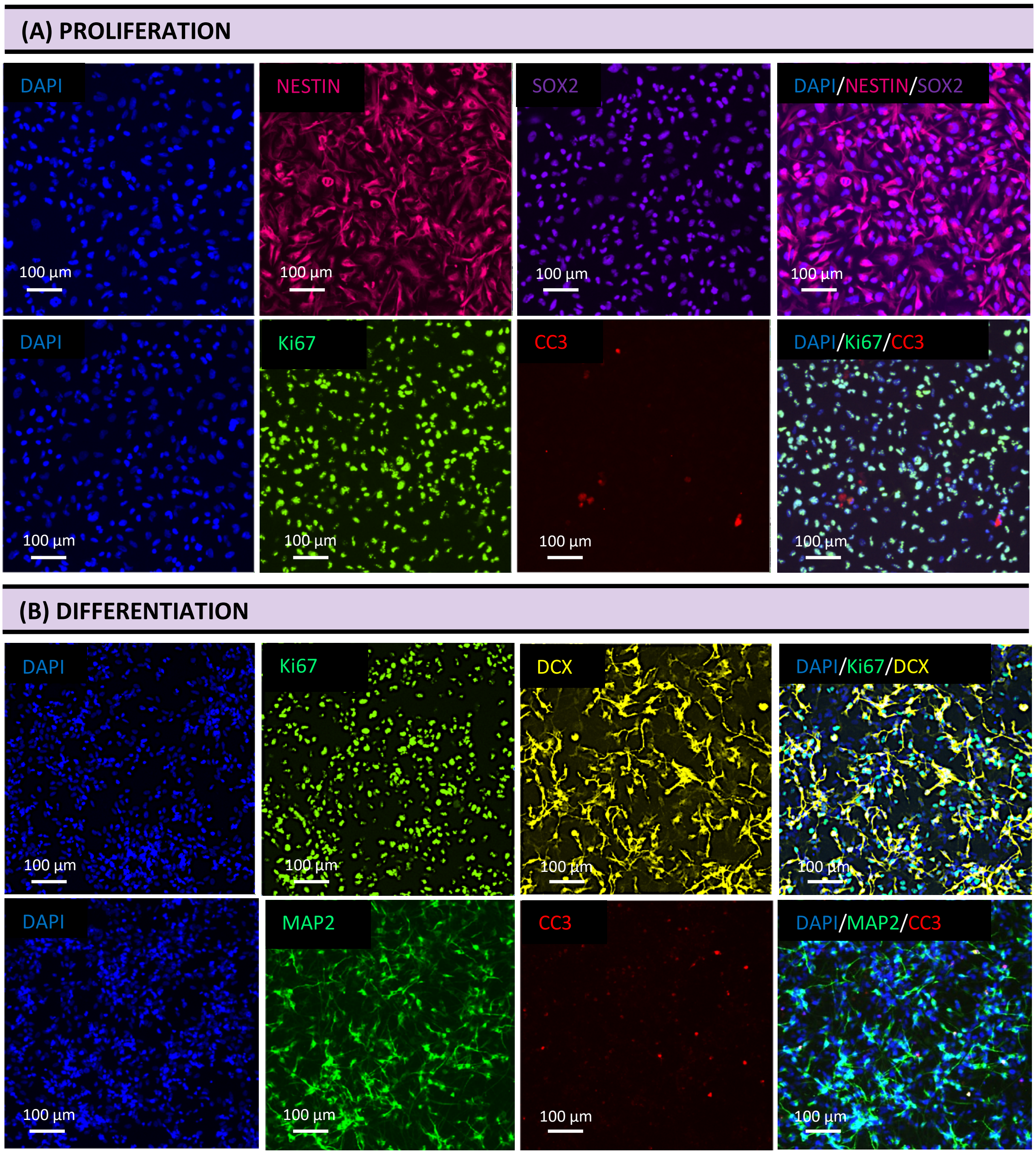

<u>Figure S.2</u> Representative images of all immunostaining.

Each box represents a field analysed by the CellInsight software. **(A) <u>Proliferation markers</u>**: Row 1 represents (from left to right) DAPI in blue, Nestin in pink, SOX2 in purple, and the composite image. Row 2 represents (from left to right) DAPI in blue, Ki67 in green, CC3 in red, and the composite image. **(B) <u>Differentiation markers</u>**: Row 1 represents (from left to right) DAPI in blue, Ki67 in green, DCX in yellow, and the composite image. Row 2 represents (from left to right) DAPI in blue, MAP2 in green, CC3 in red, and the composite image. All images taken with at 10x objective; scale bar, on the bottom left of each image, represents 100µm. Cell line: *HPC0A07/03*. <u>Abbreviations</u>: DAPI, 4',6-diamidino-2-phenylindole; CC3, cleaved caspase 3; SOX2, SRY (sex determining region Y)-box 2; DCX, doublecortin; MAP2, microtubule-associated protein 2.

segmentation parameters were modified as required to ensure nuclei were accurately outlined. DAPI stains too small or large to be counted as nuclei (i.e., DAPI- positive debris and/or unsegmented nuclei) were excluded. Moreover, nuclei touching the image field border were also excluded from further analysis.

Cellular markers of interest were identified and quantified using defined parameters that were dependent on each marker's location in relation to the nucleus. Nuclear proteins were indicated using a round target that overlaid the nuclear outline. Cytoplasmic proteins were indicated using a ring target, where the nucleus was denoted with the target's inner boundary. Average intensity thresholds (AIT) were then manually defined to distinguish specific fluorescent signals from unspecific binding and background noise. To set these, experimental wells were compared with negative control wells (i.e., unstained HPCs lacking either the primary or secondary antibody) and the threshold was set above the highest intensity signal of the negative control. Positive cells (i.e., those with an AIT above the set threshold) could then be differentiated from negative cells (i.e., those with AIT below the set threshold). These parameters were kept constant throughout every experiment. Up to fifteen fields of view per well (located in the centre of the well to ensure good coverage) were imaged. This quantified 4000–10,000 cells per well. After imaging, the software automatically calculated the percentage of all cells positive for each marker (identified by the appropriate cellular stain), as controlled for by DAPI-positive nuclei.

Neurite outgrowth

Automated neurite outgrowth analyses were performed to quantify the neurite outgrowth and branching of neuronal cells after 7 days of differentiation as previously described (27,28). Images were acquired using the CellInsight with a x10 objective as described above, and image analysis was performed using the web-based Columbus Analysis System (Perkin Elmer) using a manually created protocol. Briefly, DAPI-positive nuclei were identified using the predefined ''method C", with the parameters for thresholding, splitting and contrast settings defined to properly identify single nuclei. DAPI-positive nuclei smaller than 30μm^2 were excluded from further analysis as they were assumed to be debris. The cytoplasm was identified using the predefined ''method B" based upon DCX and MAP2 staining. Cells were

excluded from further analysis if the cytoplasm was in contact with the field edge or the average DCX/MAP2 staining intensity across the cytoplasmic area was below 120/1200 arbitrary units (AU), respectively. Only DCX/MAP2-positive neurons entirely within the field of view underwent analysis. The channels containing DCX and MAP2 subsequently underwent a sliding parabola filter step with a curvature value of 20 to remove background signal, and neurites of whole DCX/MAP2-positive cells were identified and traced using the CSIRO Neurite Analysis 2 method. Exported parameters included: total neurite length per well, maximum neurite length per well, the average number of neurite roots originating at the cell body per DCX/MAP2-positive cell, the average number of neurite segments originating at the cell body per DCX/MAP2-positive cell, the average number of neurite extremities per DCX/MAP2-positive cell and the average number of branchpoints per DCX/MAP2-positive cell.

Statistical analysis

Data analyses were conducted using SPSS Statistics 26 and R software (version 3.6.3). We first studied the association between each of the proliferation and differentiation markers and depressive symptomology reported at any point during the study (including baseline) using logistic regression. We also conducted separate logistic regression models for each depression subtype (i.e., a single occurrence, and recurrent, depressive symptomology) comparing participants with each depression subtype to those without any reported symptoms. Models were primarily adjusted for age, gender, education, and CD status, and in case of association, further adjustment was performed by including baseline depression status and potential confounders that were associated with depressive symptomology (and its chronicity) (**Table 1; Main Manuscript**). We also explored whether neurite morphology was associated with depressive symptomology, using logistic regression models as described above.

Additionally, to further explore the complicated relationship between depressive symptomology and CD (30,31), interactions were tested between each HN readout and CD in our models.

Next, for each HN marker that we found associated with depressive symptomology, we explored associations with the nutrient biomarkers, and the previously identified blood metabolites and lipids using linear regression models initially adjusted for age, gender, education, and CD. Further adjustment was performed including potential confounders associated with the HN and nutrient-related data (**Table 2, Main manuscript**).

To determine the potential mediating and/or moderating role of HN outcomes in the relationship between diet and depressive symptomology, mediation and moderation analyses were conducted using the PROCESS macro as previously described (32). Models were adjusted for age, gender, education, and CD status and for relevant potential confounders (as above).

Finally, principal component analyses (PCA) using a Promax rotation were performed, as previously described (33,34), to combine HN readouts into a neurogenic profile that we investigated in relation to depressive symptomology and chronicity.

False discovery rate correction was applied to account for multiple testing throughout and all models were bootstrapped enhanced to obtain robust estimates of standard errors.

RESULTS

Association between baseline neurogenic profiles incorporating all neurogenesis markers and depressive symptomology as stratified by chronicity

Principal component analyses revealed no differences in the overall neurogenic profiles between groups with respect to depressive symptomology and chronicity (**Figure S.3**).

(A) Neurogenic profiles for participants with and without depressive symptomology as stratified by symptom chronicity

(i) Proliferation profiles for participants as stratified by caseness for depressive symptomology

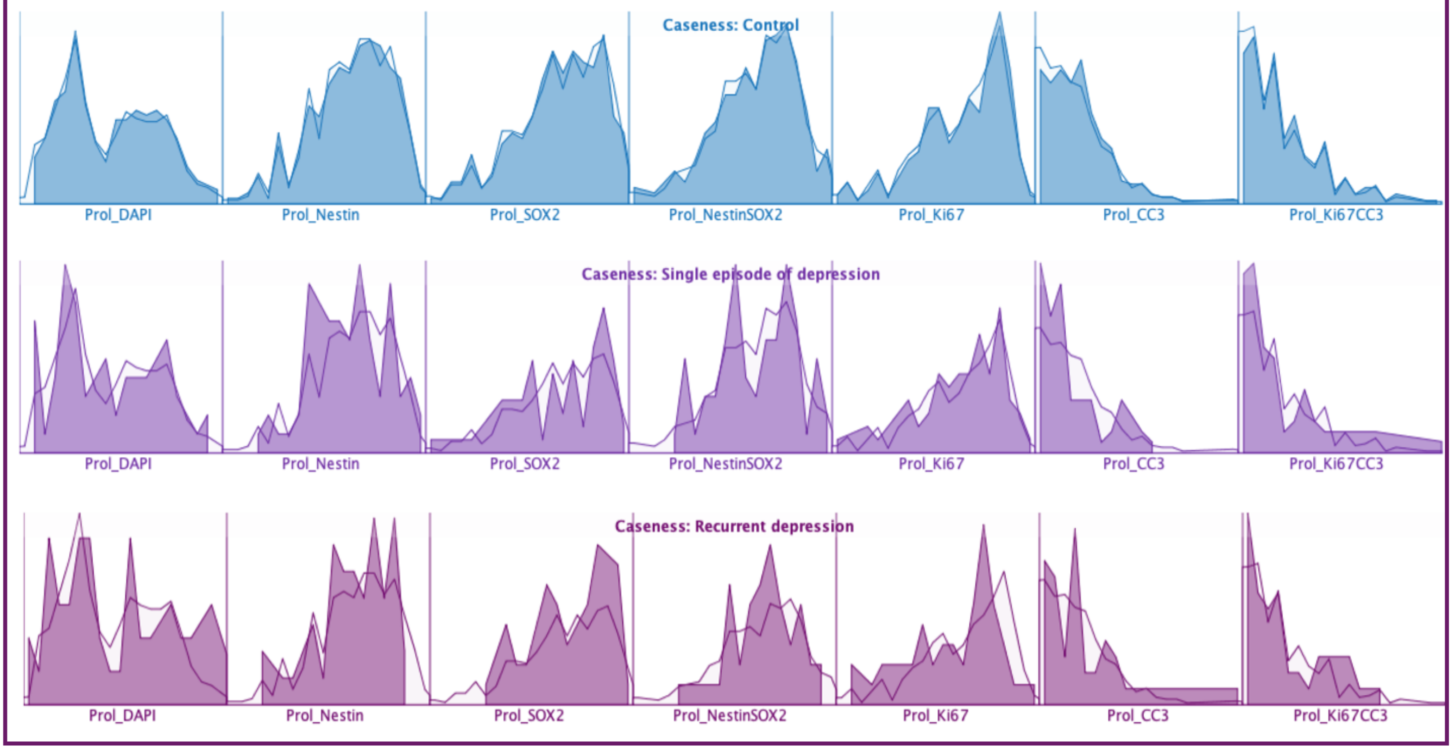

(ii) Differentiation profiles for participants as stratified by caseness for depressive symptomology

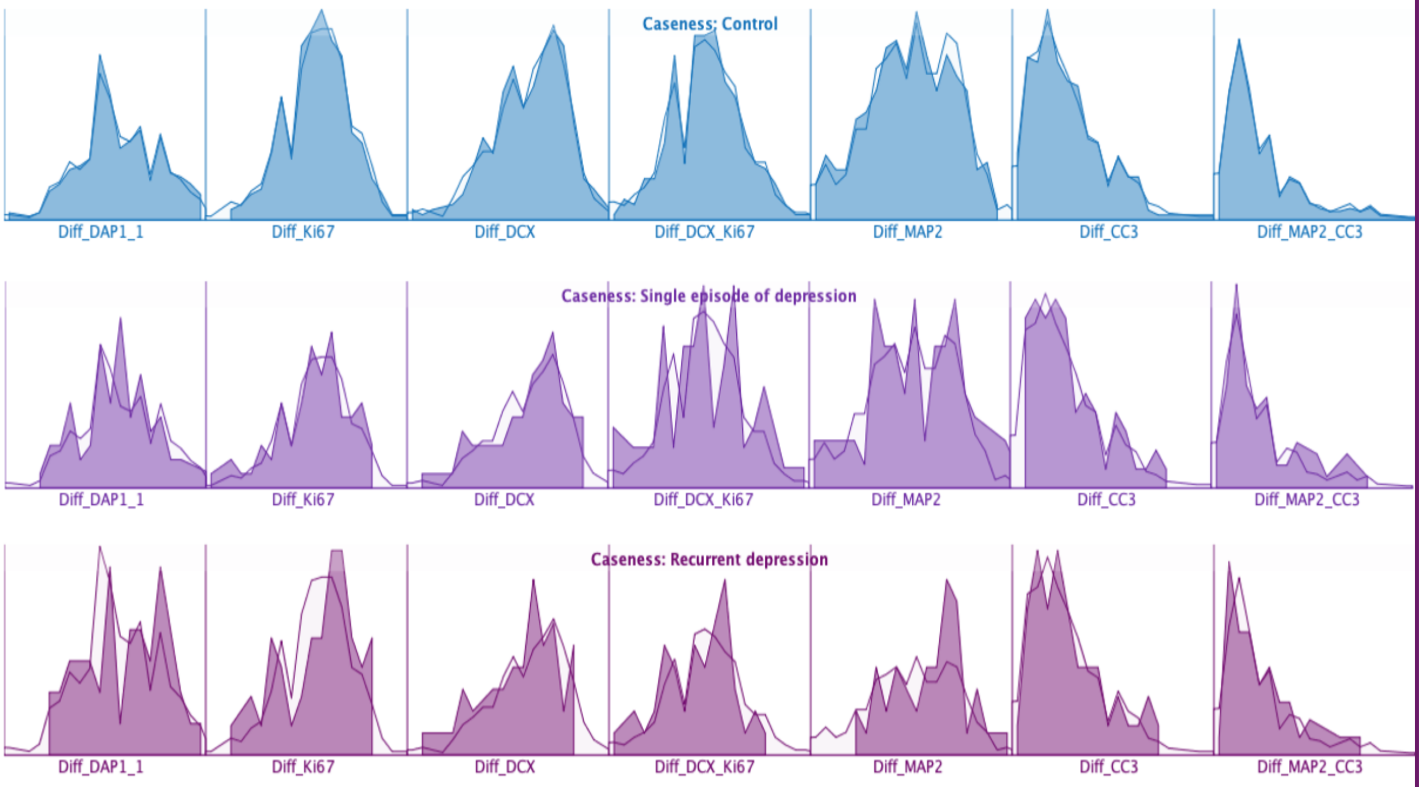

Scatter graph showing all neurogenesis markers (proliferation [PC1] by differentiation [PC2] as stratified by depressive symptom chronicity

(B)

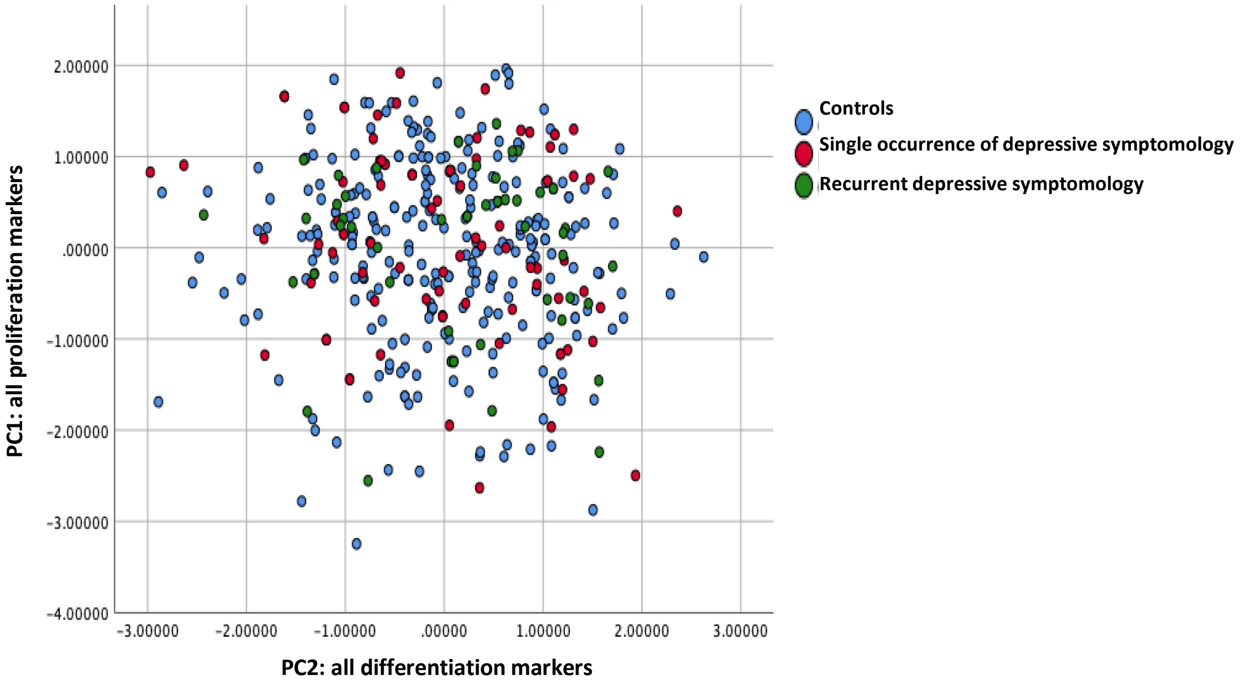

<u>Figure S.3</u>. **Association between baseline neurogenic profiles incorporating all neurogenesis markers and depressive symptomology as stratified by chronicity**

(A) Neurogenic profiles of all neurogenesis markers stratified by caseness for depressive symptomology **(i)** Representative profiles of proliferation markers in controls with no depressive symptomology (in blue) versus those having experienced a single occurrence of depressive symptomology (in lilac) and those with recurrent depressive symptomology (in aubergine). In each panel, from left to right: DAPI-, Nestin-, SOX2-, Nestin/SOX2-, Ki67-, CC3- and Ki67/CC3-positive cells levels – all at baseline. **(ii)** Representative profiles of differentiation markers in controls with no depressive symptomology (in blue) versus a single occurrence of depressive symptomology (in lilac) and recurrent depressive symptomology (in aubergine). In each panel, from left to right: DAPI-, Ki67-, DCX-, Ki67/DCX-, MAP2-, CC3- and MAP2/CC3-positive cell levels – all at baseline. **(B)** Principal component scatter graph of all proliferation markers (PC1) by all differentiation markers (PC2) stratified by caseness for depressive symptomology. No clear distinction could be made between cases with symptoms and controls when looking at the baseline neurogenic profiles (with all markers). <u>Abbreviations</u>: Prol, proliferation; Diff, differentiation; DAPI, 4′,6-diamidino-2-phenylindole; CC3, cleaved caspase 3; SOX2, SRY (sex determining region Y)-box 2; DCX, doublecortin; MAP2, microtubule-associated protein 2.

REFERENCES

1. Guertin KA, Moore SC, Sampson JN, Huang W-Y, Xiao Q, Stolzenberg-Solomon RZ, *et al.* (2014): Metabolomics in nutritional epidemiology: identifying metabolites associated with diet and quantifying their potential to uncover diet-disease relations in populations. *Am J Clin Nutr* 100: 208–217.
2. Maruszak A, Murphy T, Liu B, de Lucia C, Douiri A, Nevado AJ, *et al.* (2017): Cellular phenotyping of hippocampal progenitors exposed to patient serum predicts conversion to Alzheimer's Disease. *bioRxiv* 175604.
3. Vascular factors and risk of dementia: design of the Three-City Study and baseline characteristics of the study population. (2003): *Neuroepidemiology* 22: 316–325.
4. Schilling S, Tzourio C, Soumaré A, Kaffashian S, Dartigues JF, Ancelin ML, *et al.* (2017): Differential associations of plasma lipids with incident dementia and dementia subtypes in the 3C Study: A longitudinal, population-based prospective cohort study. *PLoS Med* 14: 1–17.
5. Naël V, Pérès K, Dartigues JF, Letenneur L, Amieva H, Arleo A, *et al.* (2019): Vision loss and 12-year risk of dementia in older adults: the 3C cohort study. *Eur J Epidemiol* 34: 141–152.
6. Low DY, Lefèvre-Arbogast S, González-Domínguez R, Urpi-Sarda M, Micheau P, Petera M, *et al.* (2019): Diet-Related Metabolites Associated with Cognitive Decline Revealed by Untargeted Metabolomics in a Prospective Cohort. *Mol Nutr Food Res* 63: 1–10.
7. Feart C, Letenneur L, Helmer C, Samieri C, Schalch W, Etheve S, *et al.* (2016): Plasma Carotenoids Are Inversely Associated With Dementia Risk in an Elderly French Cohort. *Journals Gerontol Ser A* 71: 683–688.
8. Samieri C, Féart C, Letenneur L, Dartigues J-F, Pérès K, Auriacombe S, *et al.* (2008): Low plasma eicosapentaenoic acid and depressive symptomatology are independent predictors of dementia risk. *Am J Clin Nutr* 88: 714–721.
9. Feart C, Helmer C, Merle B, Herrmann FR, Annweiler C, Dartigues J-F, *et al.* (2017): Associations of lower vitamin D concentrations with cognitive decline and long-term risk of dementia and Alzheimer's disease in older adults. *Alzheimer's Dement* 13: 1207–1216.
10. Lefèvre-Arbogast S, Gaudout D, Bensalem J, Letenneur L, Dartigues J-F, Hejblum BP, *et al.* (2018): Pattern of polyphenol intake and the long-term risk of dementia in older persons. *Neurology* 90: e1979 LP-e1988.
11. Sophie Lefèvre-Arbogast, Boris P Hejblum, Catherine Helmer, Christian Klose, Claudine Manach, Dorrain Y Low, Mireia Urpi-Sarda, Cristina Andres-Lacueva, Raúl González-Domínguez CS (n.d.): Early signature of subsequent cognitive decline in the blood lipidome of the elderly.
12. Radloff LS (1977): The CES-D Scale: A Self-Report Depression Scale for Research in the General Population. *Appl Psychol Meas* 1: 385–401.
13. BERKMAN LF, BERKMAN CS, KASL S, FREEMAN JR. DH, LEO L, OSTFELD AM, *et al.* (1986): DEPRESSIVE SYMPTOMS IN RELATION TO PHYSICAL HEALTH AND FUNCTIONING IN THE ELDERLY. *Am J Epidemiol* 124: 372–388.

14. Lyness JM, Noel TK, Cox C, King DA, Conwell Y, Caine ED (1997): Screening for Depression in Elderly Primary Care Patients: A Comparison of the Center for Epidemiologic Studies—Depression Scale and the Geriatric Depression Scale. *Arch Intern Med* 157: 449–454.
15. Radloff LS, Locke BZ, Homme D, Bjectifs O (2011): Risques psychosociaux : Center for Epidemiologic Studies-. 475–480.
16. Cosco TD, Lachance CC, Blodgett JM, Stubbs B, Co M, Veronese N, *et al.* (2020): Latent structure of the Centre for Epidemiologic Studies Depression Scale (CES-D) in older adult populations: a systematic review. *Aging Ment Health* 24: 700–704.
17. Folstein MF, Folstein SE, McHugh PR (1975): 'Mini-mental state'. A practical method for grading the cognitive state of patients for the clinician. *J Psychiatr Res* 12: 189–198.
18. Goodglass H, Kaplan E (1979): Assessment of Cognitive Deficit in the Brain-Injured Patient BT - Neuropsychology. In: Gazzaniga MS, editor. Boston, MA: Springer US, pp 3–22.
19. Isaacs B, Kennie AT (1973): The Set test as an aid to the detection of dementia in old people. *Br J Psychiatry* 123: 467–470.
20. Reitan RM (1958): Validity of the Trail Making Test as an Indicator of Organic Brain Damage. *Percept Mot Skills* 8: 271–276.
21. Anacker C, Zunszain PA, Cattaneo A, Carvalho LA, Garabedian MJ, Thuret S, *et al.* (2011): Antidepressants increase human hippocampal neurogenesis by activating the glucocorticoid receptor. *Mol Psychiatry* 16: 738–750.
22. Zunszain PA, Anacker C, Cattaneo A, Choudhury S, Musaelyan K, Myint AM, *et al.* (2012): Interleukin-1β: a new regulator of the kynurenine pathway affecting human hippocampal neurogenesis. *Neuropsychopharmacol Off Publ Am Coll Neuropsychopharmacol* 37: 939–949.
23. Anacker C, Cattaneo A, Musaelyan K, Zunszain PA, Horowitz M, Molteni R, *et al.* (2013): Role for the kinase SGK1 in stress, depression, and glucocorticoid effects on hippocampal neurogenesis. *Proc Natl Acad Sci* 110: 8708 LP – 8713.
24. Pollock K, Stroemer P, Patel S, Stevanato L, Hope A, Miljan E, *et al.* (2006): A conditionally immortal clonal stem cell line from human cortical neuroepithelium for the treatment of ischemic stroke. *Exp Neurol* 199: 143–155.
25. de Lucia C, Murphy T, Steves CJ, Dobson RJB, Proitsi P, Thuret S (2020): Lifestyle mediates the role of nutrient-sensing pathways in cognitive aging: cellular and epidemiological evidence. *Commun Biol* 3. https://doi.org/10.1038/s42003-020-0844-1
26. Borsini A, Pariante CM, Zunszain PA, Hepgul N, Russell A, Zajkowska Z, *et al.* (2019): The role of circulatory systemic environment in predicting interferon-alpha-induced depression: The neurogenic process as a potential mechanism, 2019/06/14. *Brain Behav Immun* 81: 220–227.
27. Du Preez A, Lefèvre-Arbogast S, Houghton V, de Lucia C, Low DY, Helmer C, *et al.* (2021): The serum metabolome mediates the concert of diet, exercise, and neurogenesis, determining the risk for cognitive decline and dementia. *Alzheimer's & Dementia*. pp 1–22.
28. Smeeth DM, Kourouzidou I, Duarte RRR, Powell TR, Thuret S (2020): Prolactin, Estradiol and Testosterone

Differentially Impact Human Hippocampal Neurogenesis in an In Vitro Model. *Neuroscience* 1–25.

29. Houghton V, Du Preez A, Lefèvre-Arbogast S, de Lucia C, Low DY, Urpi-Sarda M, *et al.* (2020): Caffeine Compromises Proliferation of Human Hippocampal Progenitor Cells. *Front Cell Dev Biol* 8: 806.
30. Gallagher D, Kiss A, Lanctot K, Herrmann N (2016): Depressive symptoms and cognitive decline: A longitudinal analysis of potentially modifiable risk factors in community dwelling older adults. *J Affect Disord* 190: 235–240.
31. Paterniti S, Verdier-Taillefer M-H, Dufouil C, Alpérovitch A (2002): Depressive symptoms and cognitive decline in elderly people: Longitudinal study, 2018/01/02. *Br J Psychiatry* 181: 406–410.
32. Hayes AF, Rockwood NJ (2017): Regression-based statistical mediation and moderation analysis in clinical research: Observations, recommendations, and implementation. *Behav Res Ther* 98: 39–57.
33. Principal Components Analysis (2018): *SPSS Data Analysis for Univariate, Bivariate, and Multivariate Statistics*. John Wiley & Sons, Ltd, pp 163–173.
34. Jolliffe IT (2002): Principal Component Analysis, Second Edition. *Encycl Stat Behav Sci* 30: 487.
